# The aetiology of rickets-like lower limb deformities in Malawian children

**DOI:** 10.1007/s00198-016-3541-7

**Published:** 2016-04-08

**Authors:** V. S. Braithwaite, R. Freeman, C. L. Greenwood, D. M. Summers, S. Nigdikar, C. B. D. Lavy, A. C. Offiah, N. J. Bishop, J. Cashman, A. Prentice

**Affiliations:** 1 MRC Human Nutrition Research, Elsie Widdowson Laboratory, Fulbourn Road, Cambridge, UK; 2 Department of Paediatric Orthopaedics, Robert Jones Agnes Hunt NHS Foundation Trust, Oswestry, Shropshire UK; 3 Department of Surgery, University of Cambridge, Addenbrooke’s Hospital, Cambridge, UK; 4 Nuffield Department of Orthopaedics, Rheumatology and Musculoskeletal Science, University of Oxford, Oxford, UK; 5 Department of Oncology and Metabolism, Academic Unit of Child Health, University of Sheffield, Sheffield, UK; 6 Beit Cure Orthopaedic Hospital, Blantyre, Malawi; 7 MRC Keneba, Keneba, The Gambia

**Keywords:** Africa, Blount, Children, Genu valgum, Genu varum, Phosphate, Rickets

## Abstract

**Summary:**

Debilitating rickets-like lower limb deformities are common in children throughout the world, particularly in Malawi, Africa where the causes are unknown. We have identified that Blount disease and calcium deficiency rickets are the likely causes of these deformities and propose calcium supplementation as a potential treatment of Malawian rickets.

**Introduction:**

Surgical correction of rickets-like lower limb deformities is the most common paediatric operation performed at Beit Cure Orthopaedic Hospital, Malawi. The aim of this study was to investigate the aetiology of these deformities.

**Methods:**

Children with a tibio-femoral angle of deformity >20° were enrolled (*n =* 42, 3.0–15.0 years). Anthropometric and early life and well-being data were collected. Early morning serum and urine samples were collected on the morning of the operation for markers of calcium and phosphate homeostasis. Knee radiographs were obtained, and the children were diagnosed with either Blount (BD, *n =* 22) or evidence of rickets disease (RD, *n =* 20). As BD is a mechanical rather than metabolic disease, BD were assumed to be biochemically representative of the local population and thus used as a local reference for RD.

**Results:**

There were no differences in anthropometry or early life experiences between BD and RD. Parathyroid hormone (PTH), 1,25-dihydroxyvitamin D, total alkaline phosphatase and urinary phosphate were significantly higher and serum phosphate, 25-hydroxyvitamin D (25OHD) and tubular maximal reabsorption of phosphate significantly lower in RD than BD. There was no difference in serum calcium, fibroblast growth factor 23 or markers of iron status between groups. All children had 25OHD > 25 nmol/L.

**Conclusions:**

Vitamin D deficiency is not implicated in the aetiology of RD or BD in Malawian children. The cause of RD in Malawi is likely to be dietary calcium deficiency leading to elevated PTH resulting in increased losses of phosphate from the bone and glomerular filtrate. The causes of BD remain unclear; there was no evidence in support of previously suggested risk factors such as being overweight or starting to walk early. Prior to surgical intervention, supplementation with calcium should be considered for children with RD.

## Introduction

Rickets (RD) is the most common non-communicable disease of children in the developing world [1]. At a tissue level, RD is characterised by a failure to mineralise newly formed bone in the growth plates of long bones. Rickets is accompanied by a degree of hypophosphataemia which results in impaired apoptosis of terminally differentiated chondrocytes at the growth plate leading to the characteristic metaphyseal abnormalities [2]. At a whole person level, these changes are associated with reduced growth, metaphyseal swelling and bone deformity, particularly in the lower limbs.

RD is caused by an abnormality in the way in which calcium and/or phosphate is regulated and is typically classified into one of two groups: (1) calcipaenic: a disruption in calcium metabolism typically due to vitamin D or dietary calcium deficiency and (2) phosphopaenic: a disruption of phosphate metabolism caused by excessive urinary loss of phosphate or dietary phosphate deficiency [3].

The diagnosis of RD requires appropriate radiographs, ideally both knees and wrists to show multiple growth plates, together with biochemical profiling to determine the aetiology and identify appropriate treatment strategies, i.e., dietary, pharmacological and/or surgical intervention [4]. Previous studies on the aetiology of RD in Africa have been conducted in countries such as The Gambia [5], Nigeria [6, 7] and South Africa [8], and the major cause of RD in these countries is thought to be calcium deficiency rickets caused by a low dietary calcium and high phytate diet. This results in higher concentrations of parathyroid hormone (PTH) and 1,25-dihydroxyvitamin D (1,25(OH)_2_D) as a mechanism to increase calcium concentration in the circulation. Raised alkaline phosphatase activity (TALP) is normally seen. In addition, studies of RD in The Gambia have shown that circulating phosphate concentrations are lower and urinary phosphate loss is higher in children with RD compared with local controls together with markedly elevated concentrations of fibroblast growth factor 23 (FGF23) and poor iron status [5, 9, 10]. Further studies have highlighted the inverse relationship between iron status and FGF23 and that supplementation with iron can reduce FGF23 concentration [11].

By contrast, Blount disease (BD) is a bone deformity of children which is thought to be caused by mechanical rather than metabolic abnormalities, and so, unlike RD, children with BD present with a normal biochemical profile [12]. As with RD, BD affects the lower limbs of growing children resulting in bow-leg (genu varum) deformities and is diagnosed by a radiograph of the knees. The causes of childhood BD are not clear although it has been noted to be more common in those with African ancestry [12], and some studies suggest risk factors such as starting to walk early and being overweight or obese [13]. If left untreated, RD and BD can lead to lasting disabilities, a reduction in quality of life, and in the case of RD, the underlying metabolic cause can result in poor bone growth and a propensity to fracture in later life [14].

The surgical correction, by osteotomy, of rickets-like lower limb deformities of unknown aetiology is the most common operation conducted in children at the Beit Cure Hospital in Blantyre, Malawi, East Africa with over a 100 operations performed each year. The aim of this study was to determine the aetiology of these deformities in Malawian children.

## Methods

### Participants

During 2008–2009, 77 participants with lower limb deformities between the ages of 3.0 and 15.0 years were identified at local clinics or at Beit Cure Paediatric Orthopaedic Hospital in Blantyre, Malawi, and enrolled in a surgical intervention study.

Beit Cure acts as the main elective orthopaedic hospital for Malawi, and it is consequently a tertiary referral centre. It runs a hub and spoke model running outpatient clinics (spokes) covering the majority of the country, and patients are then brought back to the hub (Beit Cure) if they require surgery. The model of health care run by Cure is free at point of delivery for all children.

Inclusion criteria for the study was being aged greater than or equal to (≥) 3 years and having a tibio-femoral angle of deformity (in degrees) ≥20. The study was described to the participants and their guardians in English and Chichewa (the local language), and written, informed consent was obtained.

### Sample collection and biochemistry

Blood and urine samples were collected in the morning prior to surgical intervention. Fresh blood samples were analysed for malaria parasites, centrifuged for haematocrit (Hct) determination, and the serum removed and stored together with the urine samples at −25 °C. The serum and urine samples were then shipped to MRC Human Nutrition Research, Cambridge, UK on dry ice where they were stored at −80 and −20 °C, respectively, until subsequent analysis.

Serum samples were analysed for 25-hydroxyvitamin D concentration (25OHD; DiaSorin Liaison, UK), PTH (Immulite, Siemens Healthcare Diagnostics, UK), 1,25(OH)_2_D (ELISA, IDS, UK) and c-terminal fibroblast growth factor 23 (C-FGF23, Immutopics Inc., CA, USA). Serum calcium (Ca), phosphate (Phos), albumin (Alb), total alkaline phosphatase (TALP), creatinine (Cr), ferritin (Ferr), c-reactive protein (CRP), cystatin C (Cys C), aspartate transaminase (AST) and urinary Ca, Phos and Cr were also measured (Kone Analyser 20i, Finland).

Assay accuracy and precision were monitored across the working range of the assays using kit controls supplied by the manufacturer. In addition, 25OHD and 1,25(OH)_2_D assay performance was monitored by DEQAS http://www.deqas.org/ and PTH by NEQAS http://www.ukneqas.org.uk.

Intra- and inter-assay coefficients of variation were <10 % for 25OHD, <5 % for all remaining analytes.

### Anthropometry, relevant history, quality of life and imaging

Height (Ht; m), sitting height (sHt; m), weight (Wt; kg) and arm span (AS; m) were measured following standard clinical procedures by trained medical staff at Beit Cure Hospital. Wt-for-age, Ht-for-age and AS-for-age *z*-scores (as a proxy for predicted Ht without limb deformity) were calculated using age and sex appropriate WHO reference data (WHO AnthroPlus v1.0.4).

A standing estimate of the tibio-femoral angle of deformity of the worst knee was measured using a goniometer. Estimated loss of height due to a lower limb deformity was calculated as the percentage difference between height and arm span measurements: $Estimated\  loss\  of\  height\ \left(\%\right) = \frac{Height(m)- Arm\  span(m)}{Height(m)}\  x\ 100$.

The guardians of the children were asked at what age the child first walked (months), whether the child had any affected relatives, whether the child was breast fed and until what age (months) and at what age the leg deformities were first noted (months). A well-being and quality of life questionnaire was administered, and the following areas were assessed: current walking ability, squatting down, playing sport, complaining of pain and being teased due to deformities. A total well-being score (TWS) of between 0 (worst) and 1 (best) was calculated from the five questions.

Radiographs of lower limbs of participants (*n =* 77) were assessed by a consultant paediatric radiologist with a special interest in metabolic bone disease. Due to the poor quality of some of the radiographs, a diagnosis was possible in only 42 of the participants. Children were diagnosed with either Blount (BD) or evidence of RD based on tibial metaphyseal (growth plate) features such as medial beaking and fragmentation (BD) and cupping and fraying (RD). None of the children showed signs of both BD and RD. There were no differences in anthropometry, physical characteristics or biochemistry between those who had radiographs of sufficient quality for diagnosis (*n* = 42) compared with those with radiographs of poor quality (*n =* 35) (data not shown).

The BD group was used as a biochemical local reference population for the RD children to be compared to. As BD is a mechanical disease and therefore should not be accompanied by a perturbed biochemistry [12], it was assumed that the BD children would have biochemical values representative of the local community.

### Statistical analysis and calculations

Haemoglobin (Hb) was calculated from Hct using the rule of 3: $H b\ \left(\frac{g}{L}\right)=\frac{Hct\ \left(\%\right)}{3}\times 10$ [15]. Ca was adjusted for Alb (Ca-corr; mmol/L) by normalising to an Alb concentration of 40 g/L using a correction factor 0.0167 mmol Ca/g albumin [16].

Glomerular filtration rate was estimated (eGFR; mL/min) using Cys C: $eGFR\ \left(\frac{mL}{min}\right)=\frac{74.835}{Cys\  C{\left(\frac{mg}{L}\right)}^{1.33}}$ [17].

Tubular maximum reabsorption of phosphate per glomerular filtration rate (TmP/GFR; mmol/L) was calculated using serum (s) and urinary (u) Phos and Cr: T $T R P=1-\left\{\left(\frac{uPhos}{sPhos}\right)\times \left(\frac{pCr}{uCr}\right)\right\}\  if\  T R P=0.86\  then\  T m P: G F R= T R P\times pPhos.\kern0.5em \mathrm{If}\  T R P>0.86\  then\  T m P: G F R=\left(\frac{0.3\times T R P}{1-\left(0.8\times T R P\right)}\right)\times sPhos$ [18].

Statistical analysis was performed using DataDesk 6.3.1 (Data Description Inc., NY, USA). Data are reported as mean and standard deviation (SD) for normally distributed data and geometric mean (−1SD, +1SD) for negatively skewed data. Group differences between children with BD vs. RD were determined using linear regression models with and without adjustment for age, sex, Wt-for-age *z*-score and group for continuous variables and chi-squared tests for binary variables. Relationships between variables were assessed using linear models with adjustment for age, sex, Wt-for-age *z*-score and group, after which an independent variable*group interaction term was included to determine group differences in the slope of the relationship between the independent and dependent variable. A probability value (*P* value) <0.05 was considered statistically significant.

## Results

### Anthropometry and early life

The children (*n =* 42) were predominantly male (67 %) and had a mean (SD) age of 5.9 (3.4) years. Sex distribution and age were not significantly different in those diagnosed with BD compared to those with RD (BD: *n* = 22, 60 % male, 6 (3) years vs. RD: *n* = 20, 75 % male, 6 (4) years, *P =* 0.3 and *P =* 0.8, respectively).

The children had a mean (SD) Wt-for-age *z*-score, Ht-for-age *z*-score and AS-for-age *z*-score of −1.1 (1.3), −3.0 (1.0) and −1.2 (1.5), a mean estimated loss of height of −8.2 (7.0) % and a mean tibio-femoral angle of the worst knee of 32° (11). None of these were significantly different by group (*P* > 0.3 for all).

Eighteen percent of BD and 35 % of RD reported having a relative with a similar lower limb deformity (group difference *P =* 0.3). The children started walking at around 19 (8) months and were breast fed until the age of 1.9 (0.7) years (group difference of *P =* 0.9 for both). Children with BD reported that their deformities appeared at a geometric mean (−1SD, +1SD) of 1.3 (0.9, 1.9) years of age compared with 1.5 (1.0, 2.0) in RD (group difference *P =* 0.4).

### Biochemistry

Children with RD had significantly lower 25OHD and higher 1,25(OH)_2_D compared with BD (Table 1). None of the children had a 25OHD concentration <25 nmol/L. Fifty percent of RD and 20 % of BD had a 25OHD < 50 nmol/L (*P =* 0.08). PTH was significantly higher in RD, and there were no group differences in serum Ca (with or without adjustment for Alb) or in urinary Ca excretion. Serum Phos and TmP/GFR were significantly lower and urinary Phos excretion significantly higher in RD compared to BD. C-FGF23 was within the normal range (<125 RU/mL) in all but one child in the RD group who had a measurement of 466.5 RU/mL, and the means did not differ significantly by group (*P =* 0.1). TALP and AST were higher in RD compared to BD children. Twenty-six percent of the children were anaemic (Hb < 110 g/L), and the mean Hb and Ferr did not differ significantly by group.

**Table 1:** Biochemistry by group

Biochemical analyte	Blount (*n* = 22)	Rickets (*n* = 20)	*P* value	
			Unadjusted	Adjusted
25-Hydroxyvitamin D (nmol/L)	72.4 (24.4)	52.2 (15.9)	0.006	0.01
1,25-Dihydroxyvitamin D (pmol/L)	239 (106)	345 (137)	0.01	0.02
Total alkaline phosphatase (U/mL)^a^	173 (113, 263)	283 (160, 499)	0.006	0.01
Phosphate (mmol/L)	1.8 (0.3)	1.5 (0.4)	0.01	0.01
Calcium (mmol/L)	2.44 (0.22)	2.33 (0.3)	0.2	0.2
Albumin (g/L)	41.5 (3.9)	39.5 (3.3)	0.09	0.2
C-fibroblast growth factor 23 (RU/mL)^a^	8.4 (6.7, 10.4)	12.2 (4.6, 32.3)	0.1	0.08
Parathyroid hormone (pg/mL)^a^	12.8 (5.9, 27.6)	30.8 (13.8, 68.8)	0.002	0.03
Cystatin C (mg/L)^a^	0.98 (0.76, 1.27)	1.02 (0.64, 1.62)	0.75	0.95
Ferritin (μg/L)^a^	44.9 (19.8, 102.0)	44.7 (20.2, 99.0)	0.9	0.8
Haemoglobin (g/L)	115 (16)	124 (16)	0.07	0.06
C-reactive protein (mg/L)^a^	6.0 (2.2, 16.4)	8.1 (2.9, 22.3)	0.4	0.2
Aspartate transaminase (U/L)^a^	4.7 (2.4, 9.3)	8.8 (3.9, 19.8)	0.01	0.1
*u*Calcium/*u*Creatinine^a^ (mmol/L)	0.03 (0.007, 0.1)	0.03 (0.007, 0.2)	0.9	0.5
*u*Phosphate/*u*Creatinine (mmol/L)	2.7 (1.6)	4.4 (2.2)	0.012	0.04
TmP/GFR (mmol/L)	1.3 (0.5)	0.8 (0.5)	0.006	0.02

Analytes refer to serum measures with the exception of two urinary (*u*) measures and tubular maximal reabsorption of phosphate per glomerular filtration rate (TmP/GFR) which is a combination of both serum and *u* measures of phosphate and creatinine. *P* values were determined by linear models, and adjusted *P* values are adjusted for age, sex and weight-for-age *z*-score. A *P* value <0.05 is considered statistically significant

^a^Biochemical analytes are presented by groups as mean (standard deviation (SD)) or geometric mean (−1SD, +1SD) for skewed variables

### Well-being questionnaire

The majority of the children reported that they had problems running and climbing, that they were in pain and that they were teased by other children due to their deformities (Table 2). This did not differ by group with the exception of being teased, which was significantly higher in BD children. There was no group difference in TWS.

**Table 2:** Well-being questionnaire

Does your child have a problem with…	Blount (*n =* 22)	Rickets (*n =* 20)	*P* value
Running and climbing (% yes)	60	75	0.3
Squatting (% yes)	20	25	0.6
Playing sport (% yes)	27	35	0.6
Pain (% yes)	68	65	0.8
Being teased (% yes)	100	75	0.01
Total well-being score (mean (SD))^a^	0.78 (0.11)	0.77 (0.12)	0.8

Well-being questions are represented as % of children who answered “yes” to experiencing any problem with the task. A total well-being score of between 0 (worst) and 1 (best) was calculated from the five questions and is presented as mean (SD). A chi-square test was used to determine group differences for categorical data. A *P* value <0.05 is considered statistically significant

^a^A two-sample Student’s *t* test was used for the total well-being score

### Predictors of TALP, Phos and tibio-femoral angle

Serum Phos was the strongest negative predictor of TALP (*r* ^2^ = 42 %, *β* coefficient (SE) = −218 (75) U/L, *P =* 0.008, Phos*group interaction *P =* 0.1), and PTH was the strongest positive predictor of TALP (*r* ^2^ = 47 %, *β* (SE) = 5.7 (1.7) U/L, *P =* 0.003, PTH*group interaction *P =* 0.2).

Serum Phos was the only significant biochemical predictor of TWS (*r* ^2^ = 27 %, *β* (SE) = 0.14 (0.05), *P =* 0.009, Phos*group interaction *P =* 0.06) and of tibio-femoral angle (*r* ^2^ = 22 %, *β* (SE) = −14° (7), *P =* 0.05, Phos*group interaction *P =* 0.07). Tibio-femoral angle significantly negatively predicted TWS (*r* ^2^ = 27 %, *β* (SE) = −0.004 (0.001), *P =* 0.005, TWS*group interaction *P =* 0.7).

## Discussion

The aim of this study was to investigate, for the first time, the aetiology of rickets-like lower limb deformities in children from Malawi, East Africa, presenting at hospital for surgery. The main findings include that the biochemical profile of Malawian children with RD is similar to what has been reported in other African studies of nutritional RD. These include elevated concentrations of PTH, 1,25(OH)_2_D, TALP and urinary Phos excretion and lower concentrations of serum Phos and TmP/GFR compared to controls which point toward a deficiency in dietary calcium. No obvious aetiological cause of BD was found in this study, and there was no evidence to support previously suggested risk factors such as early walking and being overweight or obese. In addition, in both the RD and BD children there was a higher number of males compared to females and a high proportion of children (26 %) who reported a family member (not a sibling) with similar bone deformities.

The study suggests that children with RD were excreting higher amounts of Phos in their urine, which consequently results in lower serum Phos concentration. The two main Phos regulatory hormones are PTH and FGF23 both of which cause an increase in urinary Phos loss through the internalisation of sodium-phosphate cotransporters in renal proximal tubular cells [19]. In the absence of elevated FGF23 concentration, the cause of this Phos loss is likely to be the elevated PTH concentration driven by dietary calcium deficiency. Primary vitamin D deficiency was not implicated in the aetiology of either RD or BD; all of the children had a concentration of 25OHD, the typical status marker of vitamin D, greater than 25 nmol/L. However, 25OHD concentrations were lower in the RD children, which is in keeping with reports from The Gambia and Nigeria [5, 20]. A potential mechanism for the lower 25OHD concentrations in RD is that children with calcium deficiency have greater requirements for 25OHD [21]. A chronically low dietary calcium deficiency leading to elevated PTH and 1,25(OH)_2_D may, in turn, increase the rate of hydroxylation of 25OHD to 1,25(OH)_2_D via CYP27B1 thus resulting in a greater demand and lower concentrations of 25OHD.

It is accepted that BD is more common in children of African descent; however, there is conflicting evidence as to its aetiology. Studies have hypothesised likely causes such as starting to walk early, being overweight/obese and the way in which some African infants are carried on the backs of their guardians. However, Bathfield et al., in a review of 110 South African children with Blount, showed no evidence in support of any of these proposed risk factors [12]. Furthermore, in our study, the BD children were not heavier at the time of the study nor did they report having started walking any earlier than children with RD, although we cannot say whether or not the BD children were heavier at the time of disease onset or how these data compare to healthy Malawian children.

A striking finding from this study was that 75 % of children with RD and 60 % of children with BD were male. This is unlikely a reflection of a cultural preference toward providing health care to boys over girls as there are equal numbers of boys and girls who present at the hospital for other procedures such as club foot correction (personal communication J Cashman). Moreover, this sex difference is in keeping with findings from The Gambia and Bangladesh which may point toward a propensity of boys to childhood bone disease [22, 23].

It was clear from the well-being questionnaire that the majority of children felt pain and had trouble running and climbing which we can assume was related to their deformities. Those who reported a higher well-being score had higher serum Phos and less severe deformities (as measured by tibio-femoral angle) compared with those with poorer well-being scores.

Limitations of this study include the lack of an age appropriate, healthy, local control group and the lack of available data on dietary calcium, phosphate and phytate intake. For the purpose of this study, the BD children acted as the biochemical comparative/control group for the RD children. According to the literature, BD is a mechanical disease and therefore should not be accompanied by a perturbed biochemistry [12] and so can be treated as an appropriate local biochemical control group for the RD children. Another limitation is that the children were recruited into this study on average 3 years after the initial onset of deformity, and so, the status of the children at time of onset cannot be assessed. However, the biochemical profile, which is consistent with other African children with active calcium deficiency rickets, suggests that the RD children are still showing signs of an altered metabolism. A final limitation of this study is the quality and type of radiographs available. Ideally, a clear anterior-posterior radiograph of the knee and wrist is required for the grading of rickets to take place [24]. In this study, only 54 % of participants had radiographs that were of sufficient quality to allow for diagnosis by an experienced paediatric radiologist.

In summary, surgical correction of rickets-like lower limb deformities is the most common operation performed in children at the Beit Cure Orthopaedic Hospital, Malawi. The cause of RD in this setting is likely to be dietary calcium deficiency leading to elevated PTH resulting in increased loss of Phos from the bone and glomerular filtrate. The causes of BD remain unclear; there was no evidence in support of previously suggested risk factors such as being overweight or starting to walk earlier. Vitamin D deficiency was not implicated in either RD or BD. Our findings support indirectly the view of Wesselsky et al. [3] that prior to surgical intervention, calcium supplementation should be considered for children with radiographic signs of RD.
